# The use of hyperbaric oxygen to treat actinic rectal fistula after SpaceOAR use and radiotherapy for prostate cancer: a case report

**DOI:** 10.1186/s12894-020-00767-3

**Published:** 2020-12-14

**Authors:** Tairo Kashihara, Koji Inaba, Motokiyo Komiyama, Hiroki Nakayama, Kotaro Iijima, Shie Nishioka, Hiroyuki Okamoto, Nao Kikkawa, Yuko Kubo, Satoshi Shima, Satoshi Nakamura, Ayaka Takahashi, Kana Takahashi, Kae Okuma, Naoya Murakami, Hiroshi Igaki, Yuko Nakayama, Arinobu Fukunaga, Yoshiyuki Matsui, Hiroyuki Fujimoto, Jun Itami

**Affiliations:** 1 Department of Radiation Therapy, National Cancer Center Hospital, Tsukiji 5-1-1, Chuo-ku, Tokyo, Japan; 2 Department of Urological Oncology, National Cancer Center Hospital, Tokyo, Japan; 3 Department of Radiology, National Cancer Center Hospital, Tokyo, Japan

**Keywords:** Radiotherapy, Hydrogel spacer, Side effects, Hyperbaric oxygen therapy, MR-guided radiation therapy

## Abstract

**Background:**

In definitive radiation therapy for prostate cancer, the SpaceOAR® System, a hydrogel spacer, is widely used to decrease the irradiated dose and toxicity of rectum. On the other hand, periprostatic abscesses formation and rectal perforation are known as rare adverse effects of SpaceOAR. Nevertheless, there is a lack of reports clarifying the association between aggravation of abscesses and radiation therapy, and hyperbaric oxygen therapy (HBOT) is effective for a peri-SpaceOAR abscess and rectal perforation.

**Case presentation:**

We report a case of a 78-year-old high-risk prostate cancer patient. After SpaceOAR insertion into the correct space, he started to receive external beam radiation therapy (EBRT). He developed a fever, perineal pain and frequent urination after the completion of EBRT, and the magnetic resonance imaging (MRI) revealed a peri-SpaceOAR abscess. Scheduled brachytherapy was postponed, administration of antibiotics and opioid via intravenous drip was commenced, and transperineal drainage was performed. After the alleviation of the abscess, additional EBRT instead of brachytherapy was performed with MRI-guided radiation therapy (MRgRT). On the last day of the MRgRT, perineal pain reoccurred, and MRI and colonoscopy detected the rectal perforation. He received an intravenous antibiotics drip and HBOT, and fully recovered from the rectal perforation.

**Conclusions:**

Our report indicates that EBRT can lead to a severe rectum complication by causing inflammation for patients with a peri-SpaceOAR abscess. Furthermore, HBOT was effective for the peri-SpaceOAR abscess and rectal perforation associated with EBRT.

## Background

A hydrogel spacer is used in radiation therapy (RT) for a variety of cancers to decrease the irradiated dose to the organs at risk (OARs) [1–5]. Furthermore, in pelvic radiation therapy, a hydrogel spacer is used to decrease the rectal dose [6–9]. In definitive RT for prostate cancer, The SpaceOAR® System (Boston Scientific, Marlborough, MA, USA), a hydrogel spacer, is widely used to decrease the rectal dose and toxicity [10, 11]. A prospective randomised study revealed that insertion of SpaceOAR significantly reduced the rectal dose and toxicity and improved bowel/urinary quality of life [12–14]. On the other hand, a patient who developed periprostatic abscess formation after SpaceOAR insertion was reported [15]; however, it was not clear whether SpaceOAR insertion was successful, although the infection improved after percutaneous drainage. Periprostatic abscess is a rare adverse effect of SpaceOAR, and the association between aggravation of abscesses and radiotherapy has not been clarified. Furthermore, the effectiveness of hyperbaric oxygen therapy (HBOT) for peri-SpaceOAR has not been reported. Here, we have presented a case of rectal perforation after peri-SpaceOAR abscess that was successfully treated with HBOT.

## Case presentation

**Fig. 1:**
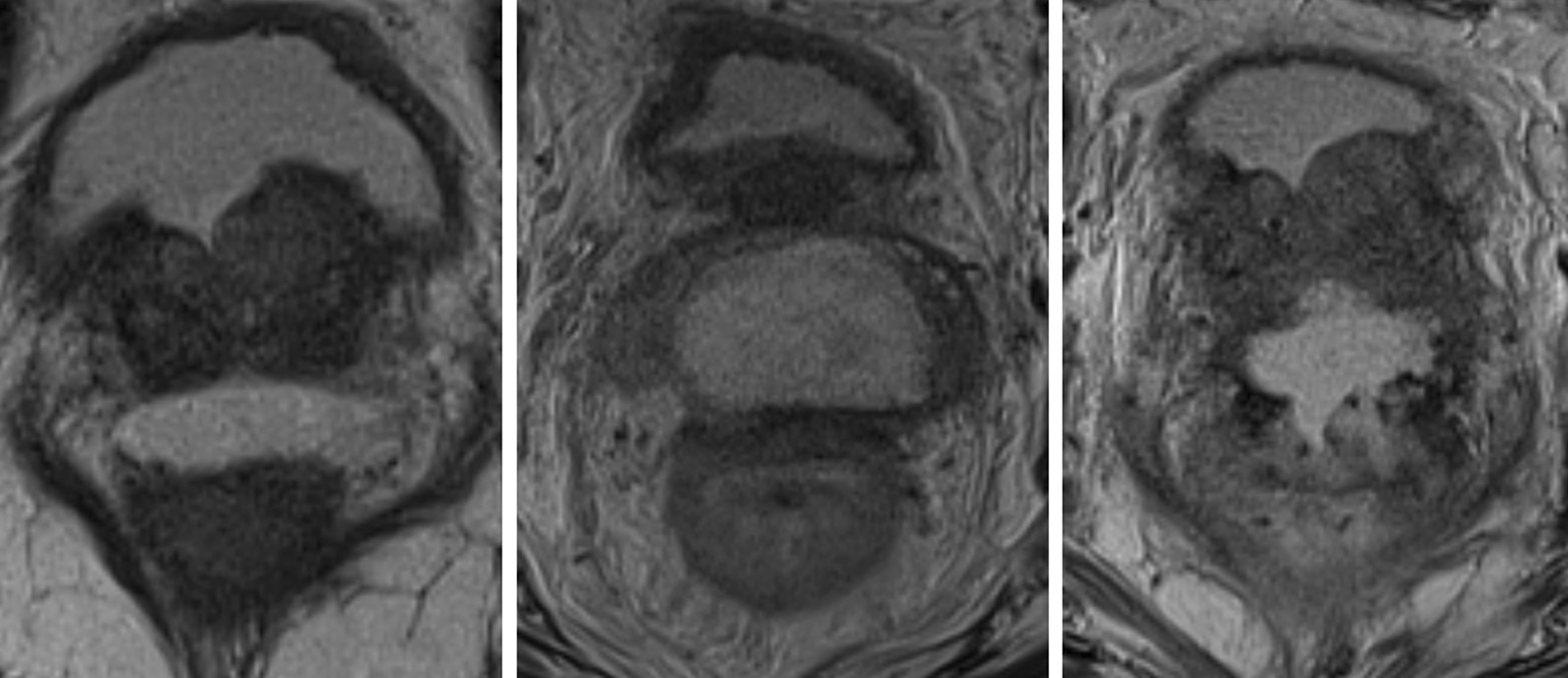
The changes of MRI findings after the SpaceOAR insertion. MRI taken 1 week after the SpaceOAR insertion (day 7, left). A peri-SpaceOAR abscess (day 61, middle) and a rectal perforation were detected on MRI (day 120, right)

**Fig. 2:**
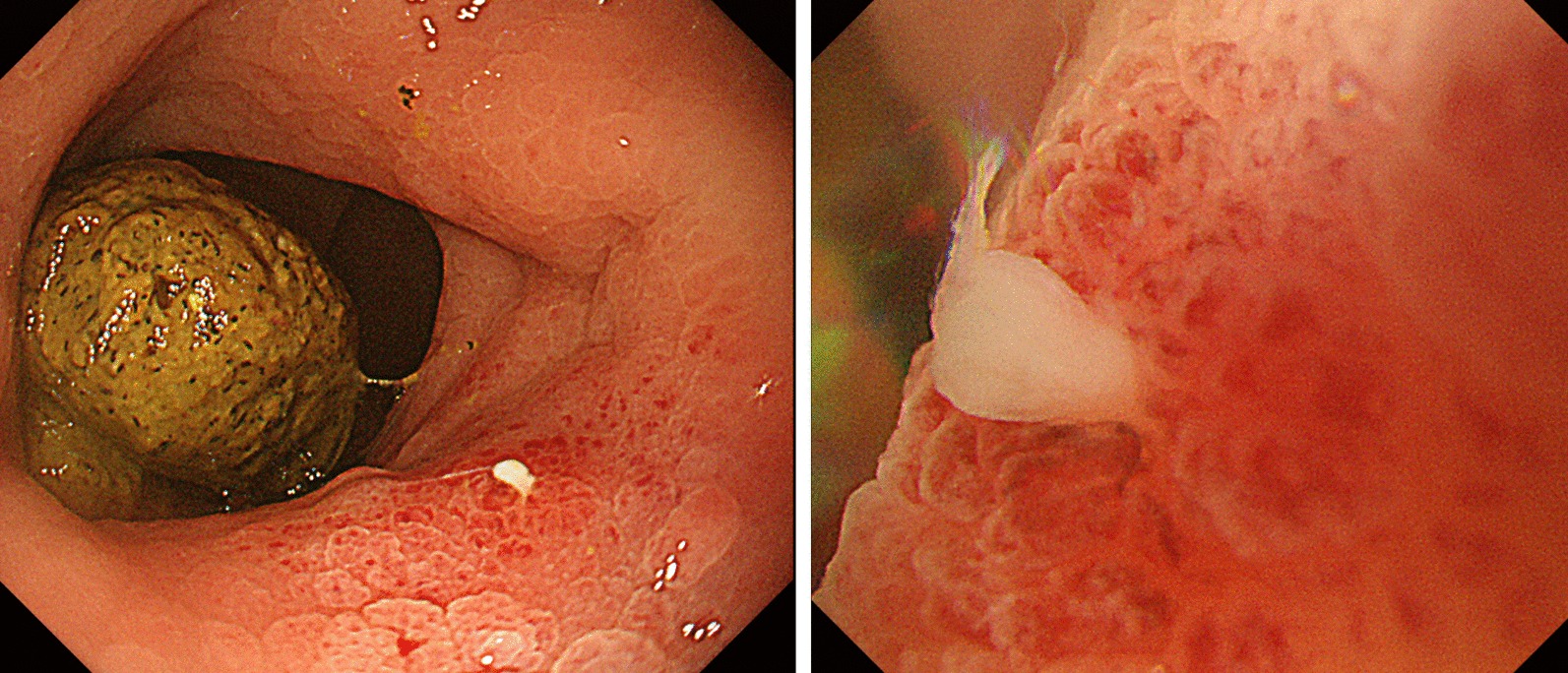
SpaceOAR penetration into rectum wall detected by colonoscopy. After the penetration of peri-SpaceOAR abscess into rectum wall was suspected on MRI, colonoscopy was performed. SpaceOAR penetrating rectum wall was detected on colonoscopy

The patient was a 78-year-old prostate cancer patient who had no medical history, except surgical history of goiter and nasal haemangioma. A prostate-specific antigen (PSA) level was 13.89 ng/mL in a routine evaluation. The clinical stage was T3a. Ultrasound-guided transperineal prostate biopsy revealed Grade Group 4 adenocarcinoma in 1 of 24 specimens. Two months after the biopsy, administration of a luteinizing hormone-releasing hormone agonist was initiated. He opted for external beam RT (EBRT) 46 Gy in 23 fractions combined with high-dose-rate (HDR) brachytherapy 15 Gy in 1 fraction as a definitive treatment. Four months after initiating hormone therapy, SpaceOAR was inserted into the space between the prostate and the rectum, and fiducial markers were inserted into the prostate under local anaesthesia with lidocaine (day 0). The insertion was completed without any side effects, and magnetic resonance imaging (MRI) confirmed that the SpaceOAR was inserted in the correct position (Fig. 1, left). Three weeks after inserting SpaceOAR (day 21), EBRT with computed tomography linear accelerator was initiated. The clinical target volume (CTV) included the prostate, all seminal vesicles, and whole pelvic lymph node regions. The planning target volume (PTV) margin of the whole pelvis was 3 mm, 7 mm, and 8 mm in the RL, SI, and AP directions, respectively. Six days after initiating EBRT (day 27), he developed perineal pain. Owing to increased perineal pain and a diagnosis of urinary tract infection on day 40, antibiotic treatment was initiated. Perineal pain gradually subsided, and he completed oral antibiotic treatment in 1 week (day 47). Four days later (day 51), he experienced perineal pain and frequent urination again; hence, antibiotic treatment was reinitiated. On day 60, oral administration of opioids was initiated due to increasing perineal pain. The next day (day 61), he developed high fever; thus, MRI was performed for detailed examination. A peri-SpaceOAR abscess was detected on MRI (Fig. 1, middle). Thus, HDR brachytherapy was postponed, administration of antibiotics and opioids via intravenous drip was initiated, and transperineal drainage was performed. Subsequently, the pain gradually subsided and the abscess shrunk slightly on MRI; therefore, intravenous administration of antibiotics changed to oral administration (day 76). Three weeks later (day 97), shrinkage of the abscess and decrease in inflammatory response were confirmed by MRI. Therefore, on day 112, additional RT was initiated. At our conference, EBRT of 20 Gy in 4 fractions was recommended as additional RT instead of HDR monotherapy to decrease the dose per fraction. Magnetic resonance-guided RT (MRgRT) with ^60^Co MRIdian under a magnetic field of 0.345 T (ViewRay MRIdian System, Oakwood Village, OH) was selected to accurately assess intrafractional and interfractional motion of the prostate, seminal vesicles, and OARs (rectum and bladder). The CTV included the prostate and seminal vesicles. The PTV margin was 5 mm, 4 mm, and 3 mm in the RL, SI, and AP directions, respectively. On the last day of MRgRT (day 119), he experienced perineal pain again, and MRI was performed. MRI on day 120 showed aggravated peri-SpaceOAR inflammation and penetration to the rectum was suspected (Fig. 1, right). Colonoscopy was subsequently performed, and penetration of SpaceOAR into the rectum was detected (Fig. 2). To treat rectal perforation, he was kept nothing per os and administration of antibiotics via intravenous drip and intravenous hyperalimentation initiated. He was also transferred to another hospital for HBOT (day 131). HBOT was initiated on day 131. After 24 HBOT sessions for 5 weeks, recovery from rectal perforation was confirmed by colonoscopy, and administration of antibiotics was discontinued. Ten weeks after termination of HBOT, disappearance of a peri-SpaceOAR abscess was confirmed on MRI (day 243).

## Discussion and conclusions

A 78-year-old patient with prostate cancer developed rectal perforation caused by a peri-SpaceOAR abscess and MRgRT (cured by HBOT). To the best of our knowledge, this is the first report showing that HBOT was effective for rectal perforation associated with a peri-SpaceOAR abscess.

Radiotherapy is one of the most significant treatment modalities in prostate cancer [16–18]. The National Comprehensive Cancer Network guidelines recommend EBRT + androgen deprivation therapy (ADT) and EBRT + brachytherapy + ADT for the treatment of high-risk prostate cancer [19]. Our patient was scheduled to receive EBRT + HDR brachytherapy + ADT. However, the treatment plan was changed to EBRT + ADT because HDR brachytherapy could cause infection [20] and a large dose per fraction could cause strong inflammation. Furthermore, MRgRT was selected because of its several potential advantages. Murray J et al. [21] reported three advantages of MRgRT—improvement in prostate visibility, monitoring of intrafractional prostate position, and daily adaptive replanning. Owing to these advantages, the margin size of MRgRT in our patient was smaller than that of CT-based RT, as mentioned above.

Radiation-induced intestinal side effects such as bleeding and ulcer are occasionally observed [22–24], but rectal perforation associated with RT is rarely observed. A case of rectal ulceration due to insertion of SpaceOAR into the anterior rectal wall was reported by Teh et al. [25]. However, in our case, SpaceOAR was inserted into the correct space between the prostate and the rectum, confirmed by MRI. A peri-prostate abscess is a rare side effect of SpaceOAR [15]. In our study, after improvement of the peri-SpaceOAR abscess, rectal perforation was detected after EBRT. Rectal perforation could have been caused by not only a peri-SpaceOAR abscess but also inflammation due to EBRT.

HBOT has been reported to be effective for the treatment of abscesses [26–29]. In addition, HBOT is also reported to be effective for the treatment of RT side effects [30–34]. We, therefore, recommended HBOT for the treatment of peri-SpaceOAR abscess and radiation-induced rectal perforation.

The exacerbation of peri-SpaceOAR abscess and rectal perforation occurred after the resumption of radiotherapy in our patient, but the abscess and rectal perforation resolved after initiating HBOT. The resumption of radiotherapy probably caused decreased blood flow and increased hypoxia, which could reduce endothelial progenitor cell (EPC) homing to the injured rectum area. However, HBOT is known to facilitate EPC trafficking/homing, thereby promoting wound repair due to angiogenesis [35, 36]. In addition, HBOT elevates hypoxia inducible factor (HIF)-1 and HIF-2 levels in vasculogenic stem/progenitor cells due to increases in reactive oxygen species [36], and this mechanism would also facilitate neovascularization. Furthermore, increasing the oxygen partial pressure can inhibit the growth of anaerobic bacteria and control the infection. HBOT may have helped our patient recover from the abscess and rectal perforation by these mechanisms.

In the management of radiation-induced haemorrhagic cystitis, early initiation of HBOT has been reported to lead to better outcomes [37]. In this previous report, HBOT within 6 months from the onset of haematuria resulted in a better response rate. In our patient, HBOT was initiated within 4 months from the onset of perineal pain, and within 2 weeks from the onset of rectal perforation. It was feared that HBOT had a cancer-promoting effect and enhanced tumour progression. However, three review articles [38–40] have reported that HBOT does not promote cancer growth. Hence, HBOT was a good treatment option in our case.

Our case report indicates that EBRT can lead to severe rectal complications by causing inflammation in patients with a peri-SpaceOAR abscess. Furthermore, it indicates that HBOT is effective for the treatment of peri-SpaceOAR abscess and rectal perforation associated with EBRT.

## Data Availability

The datasets used and/or analysed during the current study are available from the corresponding author on reasonable request.

## Abbreviations

ADT: Androgen deprivation therapy
CT: Computed tomographic
CTV: Clinical target volume
EBRT: External beam radiation therapy
HBOT: Hyperbaric oxygen therapy
HDR: High-dose-rate
HIF: Hypoxia inducible factors
LHRH: Leutinizing hormone-releasing hormone
MRgRT: Magnetic resonance imaging-guided radiation therapy
MRI: Magnetic resonance imaging
NCCN: National Comprehensive Cancer Network
OAR: Organ at risk
PTV: Planning target volume
PSA: Prostate-specific antigen
RT: Radiation therapy
